# Lung Pericytes: Molecular Mechanisms, Signaling Pathways, and Roles in Pulmonary Diseases

**DOI:** 10.1002/cph4.70205

**Published:** 2026-06-30

**Authors:** Stuti Agarwal, Anuradha Bankar, Vinicio A. de Jesus Perez

**Affiliations:** ^1^ Division of Pulmonary, Allergy, & Critical Care Medicine Stanford University Stanford California USA

## Abstract

Pericytes are specialized mural cells that ensheathe microvessels and play critical roles in maintaining vascular homeostasis, regulating angiogenesis, and coordinating tissue repair. Studies in the systemic circulation have established that pericytes contribute to the pathogenesis of major vascular diseases, including stroke, myocardial infarction, and retinopathy, increasing interest in understanding their roles in both health and disease. In contrast, our understanding of pericyte biology in the lung remains relatively limited. Over the past 15 years, a growing body of evidence emphasizes that lung pericytes actively participate in vascular remodeling and inflammatory responses, pointing to an important role for these cells in the pathogenesis of multiple pulmonary diseases. This comprehensive review synthesizes current knowledge on the molecular mechanisms governing lung pericyte function, with particular emphasis on key signaling pathways including PDGF‐BB/PDGFRβ, TGFβ/ALK1/ALK5, VEGF/VEGFR, Angiopoietin/Tie2, Notch, Wnt, and sphingosine‐1‐phosphate (S1P). We examine how these pathways orchestrate pericyte recruitment, proliferation, differentiation, and phenotypic transitions through complex downstream signaling cascades involving kinases, transcription factors, and mechanotransduction mechanisms. The review further explores the multifaceted roles of pericytes in major pulmonary diseases, including acute lung injury and acute respiratory distress syndrome (ALI/ARDS), pulmonary fibrosis, pulmonary arterial hypertension (PAH), lung cancer, and lung infections.

## Graphical abstract

Central role of pericytes across major pulmonary diseases. Conceptual wheel diagram illustrating the central role of lung pericytes across diverse pulmonary disease states. A pericyte positioned at the center expresses canonical markers, including PDGFRβ, CD146, and NG2, and connects radially to five major disease contexts. In acute lung injury and acute respiratory distress syndrome, pericyte detachment and migration contribute to microvascular barrier dysfunction, increased vascular permeability, pro‐inflammatory cytokine release, and capillary rarefaction, leading to alveolar edema and impaired gas exchange. In pulmonary fibrosis, pericytes undergo myofibroblastic differentiation and become major sources of extracellular matrix under sustained TGFβ signaling, while loss of vascular support function contributes to progressive microvascular loss. In lung cancer, tumor‐associated pericytes display abnormal recruitment and defective vessel coverage, resulting in structurally unstable and hyperpermeable tumor vasculature that facilitates tumor growth and metastatic dissemination through dysregulated VEGF and PDGF signaling. In pneumonia and lung infections, activated pericytes coordinate immune cell recruitment, regulate inflammatory vascular leakage, secrete antimicrobial mediators, and participate in tissue repair following infection resolution. In pulmonary arterial hypertension, pericyte‐to–smooth muscle cell differentiation drives progressive muscularization of small pulmonary arteries, luminal narrowing, increased pulmonary vascular resistance, and right ventricular overload through dysregulation of BMPR2, TGFβ, and hypoxia signaling pathways. Together, these disease contexts illustrate that pericytes are dynamic regulators of pulmonary vascular and tissue homeostasis whose context‐dependent dysfunction contributes to pathological remodeling across a wide spectrum of lung diseases.

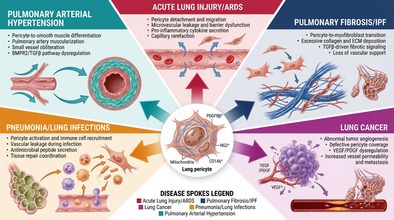

## 1 Introduction

Pericytes (PCs), specialized mural cells that intimately associate with endothelial cells along the microvasculature, have emerged as critical regulators of vascular physiology and pathophysiology. These cells not only provide mechanical support but also deliver essential paracrine cues that regulate endothelial quiescence, vascular remodeling, angiogenic sprouting, and inflammatory responses. Pericytes are widely distributed throughout the systemic and pulmonary circulatory systems and serve a multifaceted role that is aligned with the metabolic needs of the target tissue (Zang et al. 2026). In the central nervous system, pericytes are a fundamental component of the blood–brain barrier (BBB), where they display the highest degree of vascular coverage and are essential for BBB formation, structural stability, and barrier integrity (Li et al. 2025; Klouda et al. 2025; Birbrair 2019; Jiang et al. 2023; Huang 2020). As integral members of the neurovascular unit and the local vascular microenvironment, pericytes regulate multiple physiological and pathological processes through dynamic interactions with endothelial cells, astrocytes, neurons, and immune cells (Drozd et al. 2025; Gastfriend and Daneman 2026). In the retina, pericytes regulate local blood flow and capillary diameter in response to changes in the retina's metabolic demands. Pericyte loss is a major contributor to retinal diseases associated with blindness such as diabetic retinopathy and age‐related macular degeneration, in which there is retinal hemorrhage and tissue infarction resulting from weakening of retinal capillaries and inappropriate mural support (Bohler et al. 2024; D'Esposito et al. 2025; Waxman et al. 2025). In the heart, single‐nucleus transcriptomics studies have revealed that ~25% of interstitial cells are pericytes, with a pericyte‐to‐endothelial cell (EC) ratio from 1:2 to 1:3 (Avolio and Madeddu 2016; Litvinukova et al. 2020). Pericyte dysfunction and loss have been documented in cardiovascular disorders such as myocardial infarction, atherosclerosis, ischemia, and cardiomyopathy, prompting efforts to explore pericyte‐based cell therapy as treatment for these conditions (Zang et al. 2026; Maino et al. 2026; Naduthottathil et al. 2019).

In contrast to the systemic circulation, the pulmonary circulation is a low pressure, high compliance circuit that is designed to allow rapid gas exchange and transit of oxygenated blood to the left ventricle for systemic delivery. In the lung, where vessels experience unique hemodynamic tensions and gas exchange demands, pericytes exhibit remarkable heterogeneity and functional plasticity. Recent single‐cell and spatial transcriptomic studies have revealed distinct pericyte subpopulations with specialized functions in different regions of the pulmonary vasculature (Klouda et al. 2025; Travaglini et al. 2020). These advances have refined our understanding of how pericytes contribute to both physiological processes such as alveologenesis and pathological conditions including pulmonary arterial hypertension (PAH), hereditary hemorrhagic telangiectasia (HHT), and idiopathic pulmonary fibrosis (IPF) (Bordenave et al. 2020; Tual‐Chalot et al. 2020; Tanjore et al. 2009; Ricard et al. 2019).

Disruptions in pericyte function or pericyte‐endothelial communication contribute significantly to promoting vascular rarefaction, increased permeability, and pathological remodeling observed across diverse lung diseases. Understanding the molecular mechanisms that govern pericyte recruitment, proliferation, differentiation, and phenotypic transitions is therefore essential for developing targeted therapeutic strategies. This review provides a comprehensive examination of the key signaling pathways that orchestrate pericyte biology, with particular emphasis on their roles in major pulmonary diseases.

## 2 Biological Significance of Endothelial‐Pericyte Interactions in Vascular Health

**FIGURE 1:**
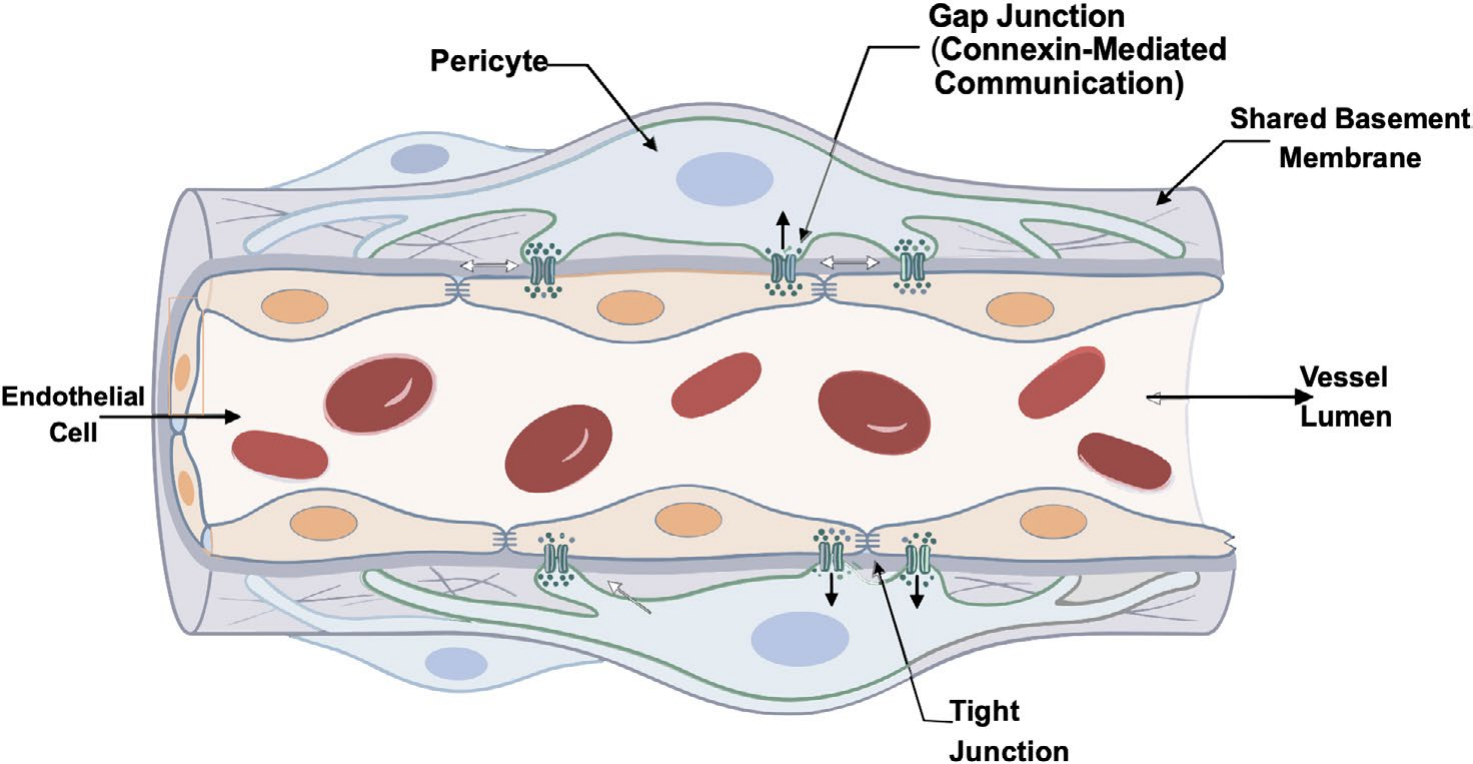
Structural and molecular mechanisms governing pericyte–endothelial cell interactions in the pulmonary microvasculature. Pericytes and endothelial cells form a highly integrated vascular unit that regulates microvascular development, stability, and barrier function. Physical interactions are mediated through direct cell–cell contacts, including peg‐and‐socket junctions, adhesion complexes, and connexin‐containing gap junctions that facilitate bidirectional communication and coordinated cellular responses. Both cell types are embedded within a shared basement membrane that provides structural support and serves as a reservoir for signaling molecules. Endothelial tight junctions maintain vascular barrier integrity, while pericytes regulate endothelial quiescence, permeability, survival, and angiogenic responses through paracrine signaling and direct contact‐dependent mechanisms. These interactions collectively control vessel maturation, blood flow regulation, extracellular matrix homeostasis, and microvascular stability under physiological conditions. Disruption of endothelial–pericyte coupling contributes to vascular leak, capillary rarefaction, inflammation, and pathological remodeling in pulmonary vascular disease.

Pericytes are abluminal mural cells that envelop capillaries and small venules, forming a structural and signaling unit with endothelial cells that underlies normal microvascular architecture and function (Klouda et al. 2025; Travaglini et al. 2020; Bordenave et al. 2020; Armulik et al. 2005; Winkler et al. 2011; Trost et al. 2016). These cells reside within the vascular basement membrane and project processes along capillaries, pre‐ and post‐capillary segments to form close physical contacts with endothelial cells (Figure 1; Tual‐Chalot et al. 2020). Pericyte processes make peg‐and‐socket and gap junction‐like contacts with endothelial cells, enabling bidirectional signaling that enforces endothelial quiescence (Klouda et al. 2025; Birbrair 2019). Furthermore, pericytes integrate into and contribute to the vascular basement membrane, linking cellular contacts to extracellular matrix structure and vessel mechanics (Li et al. 2025; Jiang et al. 2023). Pericyte coverage and associated paracrine cues suppress endothelial cell cycle activity and promote a quiescent phenotype in stable microvessels.

Pericyte–endothelial cell (EC) interactions are fundamental to vascular development, homeostasis, and repair, regulating vessel stabilization and maturation, barrier integrity and permeability, angiogenesis and vascular remodeling, microvascular blood flow, and extracellular matrix organization. In addition to regulating sprouting, pericyte presence preserves barrier function by restraining endothelial sensitivity to pro‐permeability signals in physiological settings (Klouda et al. 2025; Tual‐Chalot et al. 2020). Pericyte coverage decreases endothelial susceptibility to vascular endothelial growth factor (VEGF)‐driven permeability by maintaining junctional complexes and modulating Angiopoietin‐2 (Tie2/Ang2) signaling in intact microvessels (Sweeney et al. 2016). Beyond maintaining vascular quiescence, pericytes contribute to basement membrane deposition and remodeling, support endothelial survival, and help coordinate local perfusion through their contractile properties (Birbrair 2019; Garrison et al. 2023). In homeostatic adult vasculature, pericyte‐endothelial coupling remains a dynamic regulator of barrier competence, vascular remodeling, and microvascular stability across tissues (Gaengel et al. 2009). Through these multiple mechanisms, pericytes are essential effectors that convert nascent endothelial tubes into stable, low‐permeability vessels, ensuring the functional integrity of the microvascular network during both development and tissue homeostasis.

## 3 Molecular Signaling Pathways in Lung Pericyte Biology

**FIGURE 2:**
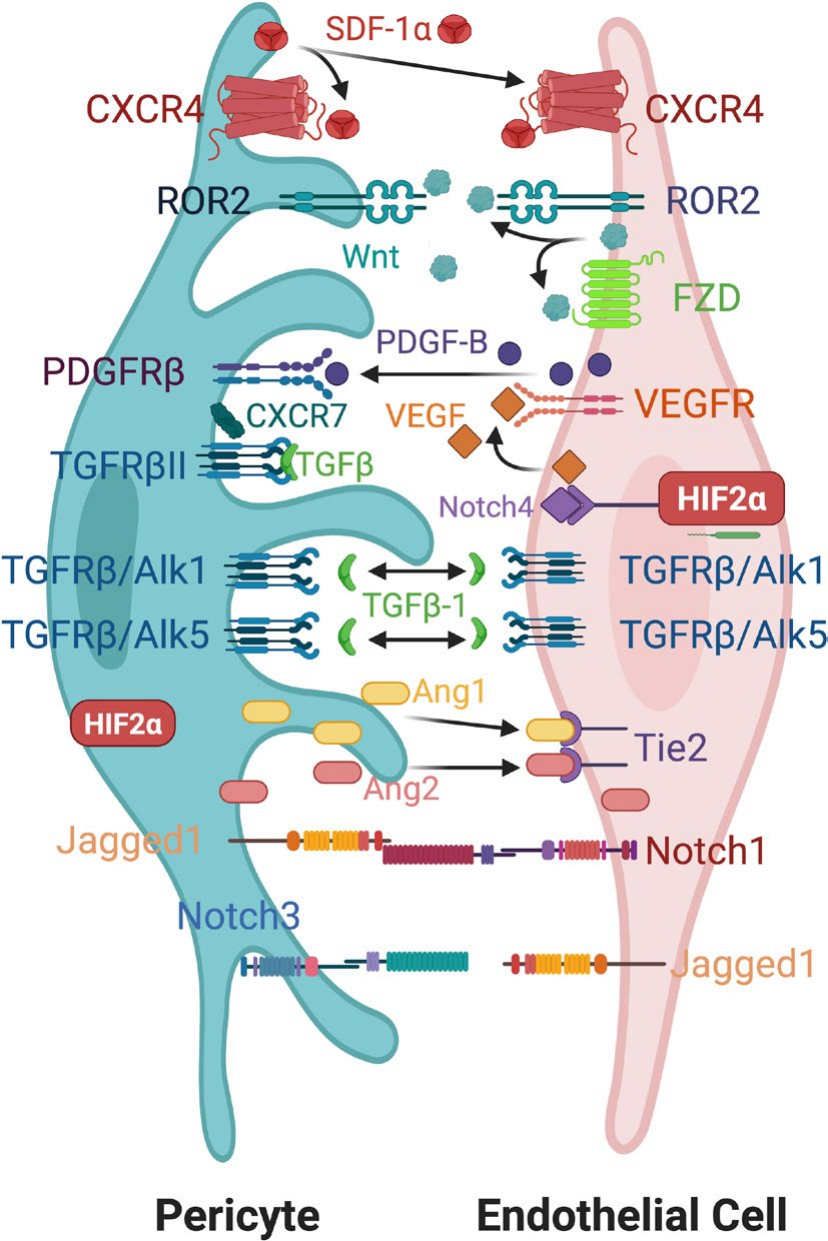
Major signaling pathways mediating pericyte–endothelial cell communication in pulmonary vascular homeostasis and remodeling. Pericytes and endothelial cells maintain vascular stability through a complex network of bidirectional signaling pathways that regulate angiogenesis, vessel maturation, barrier integrity, and cellular quiescence. Canonical PDGF‐B/PDGFRβ signaling promotes pericyte recruitment, attachment, and survival along the endothelial surface. VEGF/VEGFR signaling coordinates endothelial proliferation and angiogenic responses while influencing pericyte behavior. The Angiopoietin–Tie2 axis, consisting of stabilizing Ang1 and context‐dependent Ang2 signaling, regulates vascular maturation and endothelial barrier function. TGF‐β signaling through ALK1 and ALK5 receptors on both cell types modulates differentiation, extracellular matrix production, and vessel stabilization. Notch signaling, mediated through Jagged and Notch receptor interactions, contributes to endothelial–pericyte communication during vascular development and remodeling. Wnt signaling, including noncanonical pathways mediated by ROR2 and Frizzled (FZD) receptors, influences endothelial polarity, migration, and pericyte phenotype. Additional signaling networks, including SDF‐1α/CXCR4‐CXCR7 chemokine signaling and hypoxia‐inducible factor‐2α (HIF‐2α) pathways, integrate environmental cues such as hypoxia and inflammation to coordinate vascular adaptation. Dysregulation of these signaling axes disrupts endothelial–pericyte coupling, promoting pericyte activation, migration, phenotypic transition, and pathological pulmonary vascular remodeling in pulmonary hypertension.

The establishment of stable endothelial–pericyte (EC–PC) interactions during vascular development and remodeling is orchestrated by a tightly integrated network of paracrine, juxtracrine, and mechanotransducive signaling pathways. These pathways coordinate pericyte recruitment, adhesion, differentiation, and long‐term vessel stabilization while simultaneously regulating endothelial quiescence and barrier integrity. Figure 2 provides a graphical summary of how the pathways act across pericytes and endothelial cells.

### 3.1 PDGF‐BB/PDGFRβ Signaling

Platelet‐derived growth factor (PDGF) signaling is a core, initiating pathway in the establishment of endothelial–pericyte (EC–PC) interactions and functions primarily to recruit and position pericytes along developing blood vessels. PDGF‐BB ligands are secreted by angiogenic endothelial cells and bind to platelet derived growth factor receptor (PDGFRβ) on mural cells, driving pericyte recruitment, proliferation, and coating of nascent vessels (Li et al. 2025; Armulik et al. 2010; Hellström et al. 2001; Lindahl et al. 1997). Beyond recruitment, PDGF signaling coordinates the timing of vascular maturation by priming pericytes for subsequent differentiation signals. PDGF‐BB maintains pericytes in a motile, investment‐competent state, enabling dynamic interactions with endothelial cells before stabilization occurs. As vessels mature, attenuation of PDGF signaling allows other pathways—such as TGF‐β, Notch, and Ang–Tie2—to dominate, promoting pericyte quiescence, enhanced cell–cell adhesion, basement membrane deposition, and endothelial barrier tightening.

PDGFRβ is a receptor tyrosine kinase that, upon ligand binding, undergoes dimerization and autophosphorylation, creating docking sites for multiple downstream signaling molecules. In lung pericytes, PDGFRβ activation triggers several key signaling cascades including the Rat sarcoma (Ras)/mitogen activated protein kinase (MAPK) pathway, phosphatidylinositol 3‐kinase (PI3K)/Ak strain transforming (Akt) pathway, and phospholipase C (PLC) pathway (Wang et al. 2019). These downstream effectors coordinate pericyte proliferation, survival, migration, and attachment to the endothelium (Wilson et al. 2018; Solinc et al. 2022).

The importance of PDGF‐BB/PDGFRβ signaling is underscored by genetic studies demonstrating that loss of PDGF‐BB or PDGFRβ leads to severe deficiency in pericyte recruitment, microvascular damage, endothelial hyperplasia, and aberrant vasculature (Solinc et al. 2022). In the lung, PDGF‐BB/PDGFRβ signaling is critical for pericyte recruitment during angiogenesis and microvessel maturation (Wilson et al. 2018). In pulmonary arterial hypertension models, PDGFRβ activation contributes to increased pericyte coverage and vascular remodeling (Solinc et al. 2022). The early expression of PDGFRβ during pericyte specification highlights the fundamental importance of this pathway in pericyte formation and function (Solinc et al. 2022). In idiopathic pulmonary fibrosis, pericytes from IPF lungs migrate more rapidly and invade basement membrane matrix more readily than control pericytes in response to PDGF‐BB stimulation (Wilson et al. 2018). The PDGF‐BB/PDGFRβ pathway also plays a central role in pericyte‐to‐myofibroblast transition, a key pathological process in pulmonary fibrosis (Wang et al. 2019). Studies have shown that core fucosylation‐mediated regulation of PDGF signaling affects lung pericyte activation and fibrosis progression (Sun et al. 2019). Furthermore, the Notch1 signaling pathway promotes pericyte‐myofibroblast transition through modulation of PDGFRβ and downstream Rho‐associated, coiled‐coil‐containing protein kinase (ROCK1) signaling (Wang et al. 2019), demonstrating important pathway crosstalk.

### 3.2 TGFβ Signaling

Transforming growth factor‐β (TGF‐β) signaling is a central regulator of endothelial–pericyte (EC–PC) interactions, functioning primarily to transition vessels from a dynamic, growth‐permissive state to a stable, mature phenotype (Goumans et al. 2002). TGF‐β signaling plays a dual and context‐dependent role in pericyte biology, with distinct outcomes mediated through different receptor complexes. Unlike PDGF signaling, which initiates pericyte recruitment, TGF‐β signaling acts as a differentiation and stabilization switch that consolidates EC–PC contact, enforces cellular quiescence, and promotes long‐term vascular integrity.

TGFβ signals through two type I receptors, Anaplastic Lymphoma Kinase (ALK1 and ALK5), which exert opposing effects on endothelial cells and coordinate pericyte‐endothelial interactions during angiogenesis and vessel maturation. TGFβ ligands bind to type II receptors, which then recruit and phosphorylate type I receptors (ALK1 or ALK5). ALK1 signaling promotes endothelial proliferation and angiogenesis through activation of Smad1/5/8, while ALK5 signaling induces endothelial apoptosis and quiescence through Smad2/3 activation (Armulik et al. 2010; Goumans and Ten Dijke 2018). In pericytes, TGF‐β signaling promotes phenotypic maturation, driving expression of contractile and extracellular matrix genes while reducing migratory and proliferative behavior. This shift stabilizes pericyte attachment to the endothelium, reinforces basement membrane deposition, and strengthens mechanical coupling between the two cell types. Through these effects, TGF‐β signaling helps lock in pericyte investment and suppresses aberrant endothelial growth.

TGFβ signaling in lung pericytes drives multiple functional outcomes depending on the cellular context. In physiological conditions, TGFβ promotes pericyte maturation and vessel stabilization. However, in pathological states such as pulmonary fibrosis, sustained TGFβ signaling drives pericyte‐to‐myofibroblast transition, excessive extracellular matrix (ECM) production, and tissue fibrosis (Lin et al. 2025; He et al. 2024). This differentiation program is mediated by Smad2/3 phosphorylation and nuclear translocation, leading to transcriptional activation of pro‐fibrotic genes. Pathologically activated TGF‐β signaling induces pericyte expression of α‐smooth muscle actin, collagen, fibronectin, and other ECM components, promoting perivascular fibrosis, hypercontractility, and medial thickening. At the same time, TGF‐β suppresses pericyte migratory plasticity and alters adhesion dynamics, impairing normal EC–PC communication. TGFβ also increases VEGF levels in pericytes, fostering endothelial survival and stability while simultaneously activating myofibroblastic differentiation (Wilson et al. 2018).

Recent studies have revealed that mechanical forces play a critical role in TGFβ activation in lung pericytes. Goodwin et al. (2023) demonstrated that cyclical mechanical stretch induces TGFβ activation through mesenchymal Gαq/11 signaling. The G protein α subunits Gαq and Gα11 transmit mechanical signals to activate TGFβ2, a process that requires serine protease activity but is independent of ROCK and integrins (Goodwin et al. 2023). This mechanically activated TGFβ signaling is crucial for pericyte‐to‐myofibroblast differentiation and ECM deposition during alveologenesis (Goodwin et al. 2023). Loss of Gαq/11 signaling in pericytes results in fewer lung parenchymal myofibroblasts, reduced elastin and collagen deposition, impaired alveologenesis, and abnormal peripheral pulmonary vessels, suggesting defects in pericyte migration and differentiation (Goodwin et al. 2023). Downstream of Gαq/11‐mediated TGFβ activation, Smad2 phosphorylation serves as a key indicator of active TGFβ signaling (Goodwin et al. 2023).

Computational modeling has revealed that TGFβ‐induced endothelial cell‐pericyte decoupling can be rescued by blocking PDGF‐Rβand fibroblast growth factor‐receptor (FGF‐R) signaling with nintedanib, an approved antifibrotic therapy (Leonard‐Duke et al. 2025). This finding highlights the importance of pathway crosstalk and suggests that therapeutic strategies targeting multiple signaling nodes may be more effective than single‐pathway inhibition.

### 3.3 VEGF/VEGFR Signaling

VEGF signaling is primarily known for its role in endothelial cell biology, but emerging evidence demonstrates important functions in pericyte regulation as well. VEGF‐A, the most studied VEGF family member, signals through VEGFR1 and VEGFR2 receptors to promote neovascularization, endothelial sprouting, adhesion, migration, proliferation, and survival (Lemay et al. 2025; Olsson et al. 2006). While VEGF is predominantly produced by endothelial cells and acts in an autocrine manner, pericytes also express VEGF receptors and respond to VEGF signaling. Importantly, TGFβ treatment of human lung pericytes increases VEGF production, creating a paracrine loop that fosters endothelial survival and stability (Wilson et al. 2018). This TGFβ‐induced VEGF expression in pericytes represents an important mechanism by which pericytes support endothelial function during vessel maturation and remodeling.

VEGF also regulates endothelial junctional plasticity and basement membrane remodeling, transiently loosening endothelial–endothelial contacts and modifying the extracellular matrix to allow pericyte access to the abluminal endothelial surface (Stratman et al. 2009; Stratman and Davis 2012). High VEGF signaling maintains endothelial cells in an activated, pro‐angiogenic state that favors vessel elongation over stabilization, whereas attenuation of VEGF signaling is required for effective pericyte attachment and maturation of the vessel wall (Greenberg et al. 2008). The balance between pro‐angiogenic and anti‐angiogenic signals is critical for proper vessel formation and stabilization. Excessive VEGF signaling can lead to vascular leak and instability, while insufficient VEGF results in endothelial cell death and vessel regression (Darden et al. 2019).

### 3.4 Angiopoietin/Tie2 Signaling

The angiopoietin‐Tie2 signaling axis represents a critical pericyte‐to‐endothelial communication pathway that regulates vascular quiescence, maturation, and stability. Unlike the PDGF‐BB/PDGFRβ pathway where endothelial cells signal to pericytes, the angiopoietin system primarily involves pericyte‐derived ligands signaling to endothelial Tie2 receptors. Overall, Angiopoietin/Tie2 signaling operates as a vascular “maintenance and stress‐response axis” rather than a primary recruitment pathway. By integrating signals from pericytes, endothelial cells, and other perivascular cells, this pathway determines whether EC–PC interactions are reinforced to preserve vessel integrity or loosened to permit remodeling, angiogenesis, or pathological destabilization (Felcht et al. 2012).

Angiopoietin‐1 (Ang1) is predominantly expressed by pericytes and binds to the Tie2 receptor on endothelial cells, promoting vascular quiescence, enhancing endothelial barrier function, and providing a “brake” for leaky vessels (Suri et al. 1996; Thurston et al. 2000). Ang1/Tie2 signaling activates downstream pathways including PI3K/Akt and MAPK, which promote endothelial cell survival and inhibit vascular permeability. Through this mechanism, pericytes act as local stabilizers of the microvasculature, enforcing endothelial quiescence once vessel growth is complete. Evidence also supports cell‐autonomous roles for Ang/Tie signaling within pericytes themselves, either via low‐level Tie2 expression or through integrin‐mediated Ang1 signaling. In pericytes, Ang1 signaling promotes cell survival, limits excessive migration, and reinforces adhesive interactions with the endothelial basement membrane. These effects help maintain pericytes in a vessel‐supportive, non‐pathogenic state.

In contrast, angiopoietin‐2 (Ang2) can act as a context‐dependent Tie2 agonist or antagonist, promoting endothelial sprouting, secretion of cellular adhesion molecules, and balanced pericyte coverage (Maisonpierre et al. 1997; Fiedler et al. 2006). Elevated Ang2 destabilizes EC–PC interactions by weakening Tie2 signaling, increasing endothelial sensitivity to VEGF and inflammatory cues, and facilitating pericyte detachment or dysfunction. In disease states, including pulmonary vascular remodeling, increased Ang2 skews the Ang1/Ang2 balance toward vascular instability, impaired pericyte support, and endothelial activation.

Pulmonary pericytes exhibit crucial organ‐specific signaling properties involving the angiopoietin system. Kato and colleagues demonstrated that Yes‐associated protein (YAP)/Transcriptional co‐activator with PDZ‐binding motif (TAZ) transcriptional co‐activators in pericytes coordinate epithelial and vascular cell behavior during lung morphogenesis through regulation of hepatocyte growth factor (HGF) and angiopoietin‐1 expression (Kato et al. 2018). Pericytes produce Ang1, which activates Tie2 signaling in endothelial cells and controls HGF expression in pericytes in an autocrine fashion (Kato et al. 2018). Loss of YAP1/TAZ in pericytes impairs alveologenesis, reduces HGF and Ang1 expression, and leads to defective capillary formation in secondary septa (Kato et al. 2018). This highlights the integrated nature of pericyte signaling, where mechanical cues (substrate stiffness) influence YAP1/TAZ localization, leading to nuclear accumulation on stiff substrates and subsequent regulation of multiple growth factor pathways (Kato et al. 2018).

Pericyte‐derived Ang1 maintains endothelial integrity via Tie2 receptor signaling in endothelial cells (Wilson et al. 2018). This signaling axis is essential for maintaining the alveolar‐capillary membrane integrity and coordinating vascular repair in response to injury (Yuan et al. 2021). The Wnt/planar cell polarity pathway has also been identified as essential for pericyte recruitment during pulmonary angiogenesis (see below), working in concert with angiopoietin signaling (Wilson et al. 2018; Moss et al. 2026).

### 3.5 Notch Signaling

Notch signaling represents a highly conserved cell–cell communication pathway that plays critical roles in pericyte development, maturation, and pathological activation (Liu et al. 2010). Functionally, Notch signaling acts as a stabilizing and anti‐proliferative cue in pericytes. It suppresses excessive pericyte proliferation and plasticity while reinforcing cell–cell and cell–matrix interactions that are essential for durable EC–PC coupling. In endothelial cells, reciprocal Notch activation limits sprouting, dampens VEGF responsiveness, and promotes junctional integrity, further synchronizing endothelial quiescence with pericyte differentiation (Gridley 2010). Disruption of pericyte Notch signaling leads to impaired pericyte coverage, abnormal vessel morphology, increased permeability, and defective vascular maturation (Liu et al. 2010).

The Notch pathway is activated when Notch receptors (Notch1‐4) on one cell bind to ligands (Jagged1, Jagged2, Delta‐like 1, 3, 4) on adjacent cells (Gridley 2010; High et al. 2008). This interaction triggers proteolytic cleavage of the Notch receptor, releasing the Notch intracellular domain (NICD), which translocates to the nucleus and activates transcription of target genes. In the developing and adult vasculature, Notch receptors—particularly Notch3—are highly enriched in pericytes and vascular smooth muscle–like cells, while endothelial cells express the corresponding ligands Jagged1 and Delta‐like ligands (DLLs) (Xiang et al. 2024; Steffes et al. 2020). Engagement of endothelial ligands with pericyte Notch receptors activates canonical Notch signaling, driving transcriptional programs that promote pericyte maturation, survival, and contractile differentiation. This signaling enforces a vessel‐supportive pericyte phenotype characterized by stable adhesion, reduced migration, and enhanced structural support of the endothelium.

In the lung, Notch signaling regulates both physiological processes such as angiogenic sprouting and vessel maturation, as well as pathological processes including fibrosis and inflammation (Hellström et al. 2007; Limbourg et al. 2005). Notch1 signaling in lung pericytes promotes proliferation and differentiation toward myofibroblasts through upregulation of the PDGFRβ/ROCK1 pathway (Wang et al. 2019), in context to pulmonary fibrosis. ROCK1 works synergistically with Notch1 to mediate pericyte behavior, creating an integrated signaling network (Wang et al. 2019). PDGFRβ, acting as a downstream effector of Notch1, can modulate various signaling molecules including Ras, PI3K, and PLC (Wang et al. 2019). Notch3 signaling is particularly important for pericyte survival and maturation (Henshall et al. 2015). Peri‐arterial specification of vascular mural cells, including pericytes, requires Notch signaling, highlighting its role in establishing pericyte identity and function (Solinc et al. 2022).

In ARDS, lipopolysaccharide (LPS) exposure activates Notch signaling in lung pericytes, affecting downstream target genes and pericyte markers (Mierzejewski et al. 2024). The activation of Notch signaling during inflammatory injury supports pericyte maintenance and contributes to microvascular endothelium recovery (Mierzejewski et al. 2024). Inhibition of Notch signaling reduces pericyte markers and angiogenic factors, suggesting a protective role for Notch activation in ALI (Mierzejewski et al. 2024).

MicroRNA (miR)‐mediated regulation of Notch signaling provides an additional layer of control. Up‐regulation of miR‐146b‐5p inhibits fibrotic lung pericytes via inactivation of the Notch1/PDGFRβ/ROCK1 pathway (Shuai et al. 2022). Furthermore, exosomal miR‐107 produced by pulmonary vascular endothelial cells antagonizes profibrotic phenotypes of pericytes by targeting a pathway involving hypoxia inducible factor (HIF‐1α)/Notch1/PDGFRβ/YAP1/Twist1 (Wang et al. 2021), demonstrating complex crosstalk between hypoxia signaling, Notch, and mechanotransduction pathways.

### 3.6 Wnt Signaling

Wingless‐related integration site (Wnt) signaling pathways play multifaceted roles in pericyte biology, regulating recruitment, proliferation, differentiation, and pathological activation. The Wnt family comprises multiple ligands that signal through different receptors to activate canonical (β‐catenin‐dependent) and non‐canonical (β‐catenin‐independent) pathways. Canonical Wnt signaling is activated when Wnt ligands bind to Frizzled (Fzd) receptors and Low‐Density Lipoprotein Receptor‐Related Protein 5/6 (LRP5/6) co‐receptors, leading to stabilization and nuclear translocation of β‐catenin. In the nucleus, β‐catenin interacts with T‐cell factor/lymphoid enhancer factor (TCF/LEF) transcription factors to activate target genes involved in cell proliferation, survival, and differentiation (Nusse and Clevers 2017). In lung pericytes, Wnt/β‐catenin signaling has been implicated in both physiological and pathological processes. CD248, a pericyte‐specific marker, regulates Wnt signaling in pericytes to promote angiogenesis and tumor growth in lung cancer (Hong et al. 2022). CD248 de‐represses Wnt/β‐catenin signaling by interacting with Wnt pathway repressors Insulin‐like growth factor‐binding protein 4 (IGFBP4) and Galectin 3‐binding protein (LGALS3BP), leading to increased expression of angiogenic factors osteopontin (OPN) and Serpin peptidase inhibitor, clade E, member 1 (SERPINE1) (Hong et al. 2022). This CD248‐Wnt signaling‐angiogenic factor axis enhances pericyte proliferation and their ability to promote endothelial cell tube formation, thereby enhancing angiogenesis and lung cancer growth (Hong et al. 2022). Loss of CD248 or administration of β‐catenin inhibitors reduces tumor vessel density and functionality, suppressing angiogenesis and lung cancer growth in orthotopic models (Hong et al. 2022). These findings establish the CD248‐Wnt signaling axis in pericytes as a potential therapeutic target for lung cancer treatment.

Noncanonical Wnt signaling [e.g., Wnt5a, Wnt7a acting through planar cell polarity (PCP) pathways] is increasingly recognized as a dominant regulator of pericyte function rather than fate. Noncanonical Wnt cues control pericyte polarity, migration, cytoskeletal organization, and contractility, enabling pericytes to dynamically align with endothelial tubes and respond to biomechanical forces. These signals also modulate pericyte–EC adhesion and influence extracellular matrix remodeling, thereby shaping vessel architecture and stiffness (Wilson et al. 2018; Moss et al. 2026). Importantly, Wnt signaling intersects with other core EC–PC pathways—including PDGF, TGF‐β, Notch, and inflammatory signaling—to coordinate transitions between plastic, migratory pericyte states and stabilized, vessel‐supportive phenotypes.

In disease states such as PAH and lung fibrosis, dysregulated Wnt signaling can promote pericyte activation, metabolic reprogramming, and profibrotic behavior, contributing to endothelial dysfunction and pathological vessel remodeling. For example, loss of endothelium‐derived Wnt5a, a non‐canonical Wnt ligand, is associated with reduced pericyte recruitment and small vessel loss in PAH (Yuan et al. 2019). In pulmonary fibrosis, Wnt signaling has been identified as a key pathway driving pericyte activation and fibrotic progression (Lin et al. 2025; He et al. 2024). Reactive oxygen species (ROS)‐induced endothelial stress contributes to pulmonary fibrosis through pericytes and Wnt signaling, involving pericyte differentiation into a pathological phenotype (Andersson‐Sjöland et al. 2016). The TGFβ and Wnt signaling pathways work in concert to promote pericyte‐to‐myofibroblast transition, ECM production, and tissue fibrosis (Lin et al. 2025; He et al. 2024).

### 3.7 Sphingosine‐1‐Phosphate (S1P) Signaling

Sphingosine‐1‐phosphate (S1P) signaling functions as a key stabilizing and pro‐maturation pathway in pericytes, coordinating endothelial–pericyte (EC–PC) adhesion, cytoskeletal organization, and vascular barrier integrity. Sphingosine‐1‐phosphate (S1P) is a bioactive lipid mediator that signals through five G‐protein‐coupled receptors (S1P_1_–S1P_5_) to regulate diverse cellular processes including cell migration, proliferation, survival, and barrier function. In the vasculature, S1P signaling plays critical roles in endothelial barrier integrity and pericyte function. S1P is produced by sphingosine kinases and can act both intracellularly and as an extracellular signaling molecule. S1P_1_ is the predominant receptor on endothelial cells and promotes endothelial barrier function, cell–cell junction assembly, and vascular quiescence (Sanchez et al. 2003; Camerer et al. 2009). In pericytes, S1P signaling regulates contractility, migration, and cytoskeletal dynamics (Sanchez et al. 2003; Camerer et al. 2009). S1P signaling intersects with other pathways to coordinate vascular responses. For example, S1P_1_ signaling can modulate VEGF‐induced vascular permeability and angiogenesis. Rather than driving initial pericyte recruitment, S1P signaling primarily consolidates pericyte attachment and reinforces vessel stability once pericytes are positioned along endothelial tubes.

Insufficient S1P signaling leads to weak pericyte attachment, increased endothelial permeability, and vessel instability, whereas excessive or skewed receptor engagement (particularly toward S1PR2‐dominant signaling) can promote aberrant contractility and vascular dysfunction. In pathological settings, altered S1P gradients or receptor expression contribute to impaired EC–PC communication and maladaptive vascular remodeling. The therapeutic potential of modulating S1P signaling has been explored in various vascular diseases. S1P receptor modulators can enhance endothelial barrier function and reduce vascular leak, suggesting potential applications in treating conditions characterized by increased pulmonary vascular permeability.

### 3.8 Mechanotransduction Regulation

Beyond classical ligand‐receptor signaling, lung pericytes respond to mechanical forces and epigenetic modifications that profoundly influence their phenotype and function. These mechanisms provide additional layers of regulation that integrate environmental cues with transcriptional programs.

Mechanical forces, including substrate stiffness, shear stress, and cyclic stretch, are transduced into biochemical signals through mechanosensitive proteins and pathways. Computational models have predicted that extracellular matrix (ECM) stiffening reduces micro vessel area, along with physical uncoupling of endothelial cells and pericytes (Leonard‐Duke et al. 2025). In lung pericytes, substrate stiffness influences YAP1/TAZ localization, with nuclear accumulation occurring on stiff substrates (Kato et al. 2018). This mechanosensitive regulation of YAP/TAZ is critical for pericyte function during lung morphogenesis and maintaining EC‐PC homeostasis in fibrotic disease (Leonard‐Duke et al. 2025). In idiopathic pulmonary fibrosis, human pericytes adopt myofibroblast properties in the fibrotic lung microenvironment, with the fibrotic ECM facilitating mechanoresponsive expression of α‐ smooth muscle actin (α‐SMA) (Sava, Ramanathan, Dobronyi, et al. 2017). Increased substrate stiffness induces megakaryoblastic leukemia 1 (MKL1)‐dependent α‐SMA expression in pericytes, creating a feed‐forward loop where TGFβ1 activates pericytes to produce ECM and increase tissue stiffness, further facilitating α‐SMA^+^ pericyte emergence via MKL‐1/Myocardin‐Related Transcription Factor A (MRTFA) mechanotransduction (Sava, Ramanathan, Dobronyi, et al. 2017).

Integrin‐mediated mechanotransduction also plays a critical role in pericyte phenotypic transitions. In bleomycin‐induced lung injury, Myh11 lineage‐positive pericytes show increased αv integrin expression, and αvβ3 integrin engagement on fibronectin drives pericyte‐to‐myofibroblast transition (Hannan et al. 2021). Blocking αvβ3 binding prevents expression of the myofibroblast marker α‐SMA, suggesting that this matrix‐integrin axis represents a potential therapeutic target (Hannan et al. 2021).

### 3.9 Epigenetic and Post‐Translational Regulation

Epigenetic modifications, including DNA methylation, histone modifications, and non‐coding RNA regulation, provide stable yet reversible control of gene expression in pericytes. Long non‐coding RNA GAS5 suppresses TGFβ1‐induced transformation of pulmonary pericytes into myofibroblasts by recruiting the histone demethylase KDM5B and promoting H3K4me2/3 demethylation of the PDGFRα/β promoter (Wang et al. 2023). GAS5 overexpression attenuates lung fibrosis in mice, identifying it as a potential intervention target for IPF (Wang et al. 2023).

MicroRNAs also regulate pericyte phenotype and function. As mentioned previously, miR‐146b‐5p inhibits fibrotic lung pericytes via inactivation of the Notch1/PDGFRβ/ROCK1 pathway (Shuai et al. 2022), while exosomal miR‐107 from endothelial cells antagonizes profibrotic phenotypes by targeting the HIF‐1α/Notch1/PDGFRβ/YAP1/Twist1 axis (Wang et al. 2021). These findings highlight the importance of intercellular communication through extracellular vesicles and microRNAs in regulating pericyte behavior.

Core fucosylation, a post‐translational modification of glycoproteins, also regulates multiple signaling pathways in lung pericytes. Knockdown of fucosyltransferase 8 (FUT8), the enzyme responsible for core fucosylation, significantly blocks lung pericyte activation and pulmonary fibrosis progression, affecting both PDGF and TGFβ pathways (Sun et al. 2019).

## 4 Pericytes in Lung Diseases

Pericytes are essential regulators of microvascular integrity, and disruption of their normal function can lead to profound vascular dysfunction characterized by impaired perfusion, tissue ischemia, and progressive organ injury. Evidence from the systemic circulation indicates that pericytes are highly dynamic participants in vascular injury and repair. In the setting of brain ischemia, myocardial infarction, retinal hemorrhage, and ischemia–reperfusion injury, pericytes become activated, detach from the microvascular wall, and adopt pro‐inflammatory phenotypes that influence endothelial behavior and local immune responses (Zang et al. 2026; Drozd et al. 2025; Bohler et al. 2024; D'Esposito et al. 2025; Waxman et al. 2025; Avolio and Madeddu 2016; Maino et al. 2026; Naduthottathil et al. 2019). In myocardial infarction, activated pericytes may also undergo phenotypic transitions toward profibrotic states, contributing to vascular remodeling and vasculopathy (Pham et al. 2021; Alex and Frangogiannis 2019; Alex et al. 2023). Although the biology of pericytes in the lung is only beginning to be defined, emerging studies suggest that similar processes occur during pulmonary vascular and parenchymal injury. The following sections summarize current evidence implicating pericytes in a range of lung disorders, with the goal of providing a conceptual framework for understanding disease mechanisms and identifying potential therapeutic strategies based on modulation of pericyte biology. Potential therapeutic implications for targeting the pathways discussed in the previous section are summarized in Table 1.

**TABLE 1:** Therapeutic strategies targeting pericytes in lung disorders.

Disease/Condition	Therapeutic strategy/Agent	Mechanism of action/Pericyte target
ALI and ARDS	S1P (Sphingosine‐1‐phosphate)	Attenuates pericyte loss, reduces vascular hyperpermeability, and preserves VE‐cadherin/N‐cadherin contacts
	Notch signaling modulation	Regulates pericyte activation and angiogenic factor expression to support endothelial recovery
	ANGPTL4 inhibition	Hypothesized to promote endothelial integrity and decrease microvascular leak during injury and resolution
Pulmonary fibrosis (PF/IPF)	Nintedanib	Tyrosine kinase inhibitor; promotes MMP production/activation in pericytes to reverse the fibrotic phenotype and soften the matrix
	miR‐146b‐5p upregulation	Inactivates the Notch1/PDGFRβ/ROCK1 pathway to inhibit the transition of pericytes into myofibroblasts
	GAS5 overexpression	Long non‐coding RNA that recruits KDM5B to demethylate the PDGFRα/β promoter, suppressing myofibroblast transformation
	αV\ß3 integrin blocking	Prevents matrix‐integrin engagement on fibronectin, inhibiting the expression of the myofibroblast marker αSMA.
	Notch1 inhibition	Suppresses pericyte proliferation and differentiation into matrix‐producing myofibroblasts.
Pulmonary arterial hypertension (PAH)	HIF2 α inhibition	Reduces pericyte migration, contractility, and transformation into smooth muscle‐like cells.
	CD13 blockade (B‐CD13 peptide)	Reverts high proliferation rates and attenuates vascular remodeling.
	CNP/cGMP/FoxO3 pathway	Augmentation prevents pericyte proliferation and transdifferentiation by stabilizing FoxO3.
	Wnt/PCP pathway restoration	Supplementation of Wnt5a or Fzd7/cdc42 to improve pericyte‐endothelial interactions and prevent vessel loss.
	CXCR7/CXCR4 inhibition	Mitigates pathological pericyte recruitment and expansion in early stages of remodeling.
Lung cancer	CD248/ß‐catenin targeting	Inhibits the CD248‐Wnt axis to reduce expression of angiogenic factors (OPN, SERPINE1) and impair tumor vessel growth.
	CCL28/RA/RXRα/ANGPT1	Modulating this pathway promotes “vascular normalization,” potentially improving the delivery of chemo‐ and immunotherapy.
	PD‐L1 blockade	Targets increased PD‐L1 expression on tumor‐associated pericytes to counter immune evasion.
	IL‐6 inhibition	Targets pericyte‐derived inflammatory signals that support the tumor microenvironment.
Pneumonia & sepsis	Fli‐1 inhibition	Prevents pericyte pyroptosis (inflammatory death), reduces vascular leak, and inhibits cytokine production.
	ANGPTL4 inhibition	(In viral injury) Promotes endothelial integrity and limits pathological vascular permeability.
	TLR modulation	Targeting TLR2, 4, and 6 to manage the inflammatory chemokine response triggered by pathogens.

## 5 Acute Lung Injury and Acute Respiratory Distress Syndrome

Lung pericytes have emerged as critical regulators of acute lung injury (ALI) and acute respiratory distress syndrome (ARDS) pathogenesis (Mierzejewski et al. 2024; Zhou and Huang 2024). In the context of ALI/ARDS, pericytes play multifaceted roles in regulating microvascular permeability, a hallmark feature of these conditions. Studies have demonstrated that pericyte dysfunction contributes to increased vascular leak through disruption of pericyte‐endothelial communication (Yuan et al. 2021; Mierzejewski et al. 2024). The activation of Notch signaling in lung pericytes during inflammatory injury supports their maintenance and contributes to microvascular endothelium recovery, with inhibition of this pathway reducing pericyte markers and angiogenic factors (Mierzejewski et al. 2024).

Pericytes possess surprising cellular plasticity, behaving dynamically when activated and participating in divergent host responses to injury (Rayner et al. 2023). Their involvement extends beyond structural support to active participation in the immune inflammatory response, influencing the progression of ALI through various signaling cascades (Zhou and Huang 2024). Recent evidence suggests that pericytes may drive vascular inflammation during viral infections, including SARS‐CoV‐2, highlighting their role in pandemic‐related lung pathology (Rayner et al. 2023). Pericytes respond dynamically to inflammatory stimuli, mediating inflammation through leukocyte trafficking and cytokine signaling while responding to pathogen‐associated molecular patterns (PAMPs) and damage‐associated molecular patterns (DAMPs) (Rayner et al. 2023). This dual role positions pericytes as both structural guardians of vascular integrity and active participants in the inflammatory response.

In experimental viral lung injury, lung pericytes demonstrate significant angiogenic activity that influences vascular permeability and endothelial integrity (Hung and Altemeier 2022). ANGPTL4 secreted by pericytes affects endothelial cell behavior, and knockdown of ANGPTL4 is hypothesized to be protective in acute viral lung injury by promoting endothelial integrity and decreasing microvascular leak during both injury and resolution phases (Hung and Altemeier 2022). This mechanism highlights the dual nature of pericyte responses during infection, where their activation can contribute to both pathological vascular leak and subsequent repair processes.

### 5.1 Therapeutic Targeting

Multiple studies have identified pericytes as effective therapeutic targets for ALI/ARDS treatment (Table 1), with potential interventions aimed at preserving pericyte‐endothelial interactions and preventing pericyte loss (Mierzejewski et al. 2024; Zhou and Huang 2024). Modulation of Notch signaling in pericytes during inflammatory injury presents one potential therapeutic strategy, as inhibition of this pathway affects pericyte activation and angiogenic factor expression (Mierzejewski et al. 2024). Pericyte retention and survival S1P administration attenuated LPS‐induced pericyte loss, reduced vascular hyperpermeability, and improved survival in murine sepsis models; protection correlated with preserved VE‐cadherin and N‐cadherin deposition at contacts. Future research dissecting the complex interactions of pericytes with other pulmonary cell populations will likely reveal additional therapeutic opportunities for these devastating lung disorders (Yuan et al. 2021; Abdel Rahman 2020).

## 6 Pulmonary Fibrosis

Pericytes play a pivotal role in the pathogenesis of pulmonary fibrosis (PF) and idiopathic pulmonary fibrosis (IPF), serving as key cellular mediators of fibrotic remodeling through their capacity to transdifferentiate into myofibroblasts. The presence and activation of pericytes lead to inflammation and fibrosis in the lung interstitium and alveolar space through the release of various cytokines and chemokines, while simultaneously stimulating fibroblast proliferation and activation to promote PF progression (Lin et al. 2025; He et al. 2024).

The transdifferentiation of pericytes into myofibroblasts represents a central mechanism in pulmonary fibrosis pathogenesis. Human pericytes adopt myofibroblast properties in the microenvironment of the IPF lung, increasing tissue stiffness by producing fibrotic ECM and high expression of α‐SMA (Sava, Ramanathan, Dobronyi, et al. 2017). Increased substrate stiffness induces MKL1‐dependent α‐SMA expression in pericytes, creating a feed‐forward loop where TGFβ1 activates pericytes to produce ECM and increase tissue stiffness, further facilitating α‐SMA^+^ pericyte emergence mechanotransduction (Sava, Ramanathan, Dobronyi, et al. 2017).

This pericyte‐to‐myofibroblast transition is regulated by multiple signaling pathways, most notably the TGFβ, MKL‐1/MRTFA, and Wnt signaling cascades (Lin et al. 2025; He et al. 2024). The Notch1 pathway also promotes this transition through the PDGFRβ/ROCK1 signal pathway, with studies demonstrating that Notch1 activation drives pericyte conversion to matrix‐producing myofibroblasts (Wang et al. 2019). Conversely, knockdown of PDGFRβ or ROCK1 suppresses pericyte proliferation and differentiation (Wang et al. 2019). Inhibition of Notch1 in mouse models of lung fibrosis alleviates fibrosis by suppressing pericyte proliferation and differentiation and inhibiting the PDGFRβ/ROCK1 pathway (Wang et al. 2019).

### 6.1 Molecular Regulation and Epigenetic Control

Molecular regulation of pericyte transdifferentiation involves complex epigenetic and signaling mechanisms. Long non‐coding RNA GAS5 suppresses TGFβ1‐induced transformation of pulmonary pericytes into myofibroblasts by recruiting KDM5B and promoting H3K4me2/3 demethylation of the PDGFRα/β promoter, with GAS5 overexpression attenuating lung fibrosis in mice (Wang et al. 2023). This epigenetic mechanism provides a novel layer of control over pericyte phenotype and represents a potential therapeutic target. Up‐regulation of miR‐146b‐5p inhibits fibrotic lung pericytes via inactivation of the Notch1/PDGFRβ/ROCK1 pathway, presenting another potential therapeutic strategy (Shuai et al. 2022). The identification of these regulatory mechanisms provides multiple intervention points for modulating pericyte behavior in fibrotic lung disease.

Additionally, ROS‐induced endothelial stress contributes to pulmonary fibrosis through pericytes and Wnt signaling, involving pericyte differentiation into a pathological phenotype (Andersson‐Sjöland et al. 2016). Recent research has identified β‐III tubulin as a marker of an anti‐fibrotic state of pericytes, suggesting heterogeneity in pericyte populations and their functional states during fibrosis (Sato et al. 2025). This finding highlights the importance of understanding pericyte subpopulations and their distinct contributions to fibrotic progression.

### 6.2 Mechanotransduction and Matrix Interactions

The mechanical properties of the fibrotic lung microenvironment profoundly influence pericyte phenotype. In bleomycin‐induced lung injury, Myh11 lineage‐positive pericytes show significantly increased contractile and secretory markers, along with αv integrin expression (Hannan et al. 2021). These pericytes undergo transition into myofibroblastic phenotypes, a process driven by αvβ3 integrin engagement on fibronectin (Hannan et al. 2021). Blocking αvβ3 binding prevents the expression of the myofibroblastic marker αSMA, suggesting that this matrix‐integrin axis contributes to pericyte‐to‐myofibroblastic transition and represents a potential therapeutic target (Hannan et al. 2021).

The importance of mechanotransduction is further demonstrated by studies showing that substrate stiffness influences YAP/TAZ localization in pericytes (Kato et al. 2018). This mechanosensitive regulation creates a pathological feed‐forward loop in fibrosis, where increased matrix stiffness drives further pericyte activation and ECM production (Leonard‐Duke et al. 2025).

### 6.3 Therapeutic Targeting

Therapeutic targeting of pericytes offers promising avenues for pulmonary fibrosis treatment (Table 1). Nintedanib, a tyrosine kinase inhibitor approved for IPF, reverses the α‐SMA^+^ phenotype and elastic modulus of fibrotic lung matrices by promoting matrix metalloproteinase (MMP) production and activation in pericytes, suggesting a novel mode of action for this antifibrotic therapy (Sava, Ramanathan, Dobronyi, et al. 2017). This finding indicates that nintedanib may work in part by modulating the mechanical properties of the fibrotic matrix through effects on pericyte‐derived MMPs.

The identification of GAS5 as an intervention target for IPF, along with strategies to modulate mechanosensitive signaling in pericyte‐derived myofibroblasts, provides multiple therapeutic opportunities (Wang et al. 2023). Targeting the Notch1/PDGFRβ/ROCK1 pathway through miR‐146b‐5p upregulation or direct pathway inhibition represents another promising approach (Shuai et al. 2022). Inhibition of Notch1 in mouse models of lung fibrosis alleviates fibrosis by suppressing pericyte proliferation and differentiation (Wang et al. 2019).

These pericyte‐targeted approaches represent novel strategies for treating patients with this debilitating lung disease (Lin et al. 2025; He et al. 2024). Future therapeutic development will likely focus on combination strategies that target multiple aspects of pericyte biology, including signaling pathways, mechanotransduction, and epigenetic regulation to more effectively prevent or reverse pulmonary fibrosis.

## 7 Pulmonary Arterial Hypertension

Pulmonary arterial hypertension (PAH) is a progressive vascular disease characterized by extensive remodeling of the pulmonary vasculature, leading to increased pulmonary vascular resistance and right ventricular failure. While endothelial cells and smooth muscle cells have traditionally been considered the primary cellular drivers, emerging evidence reveals that pericytes—mural cells residing at the abluminal surface of capillaries and small vessels—play a critical role in pulmonary vascular remodeling. Recent lineage tracing studies, single‐cell transcriptomics, and functional investigations have transformed our understanding of pericyte biology in PAH.

### 7.1 Pericyte Involvement in PAH Pathogenesis (Figure 3)

**FIGURE 3:**
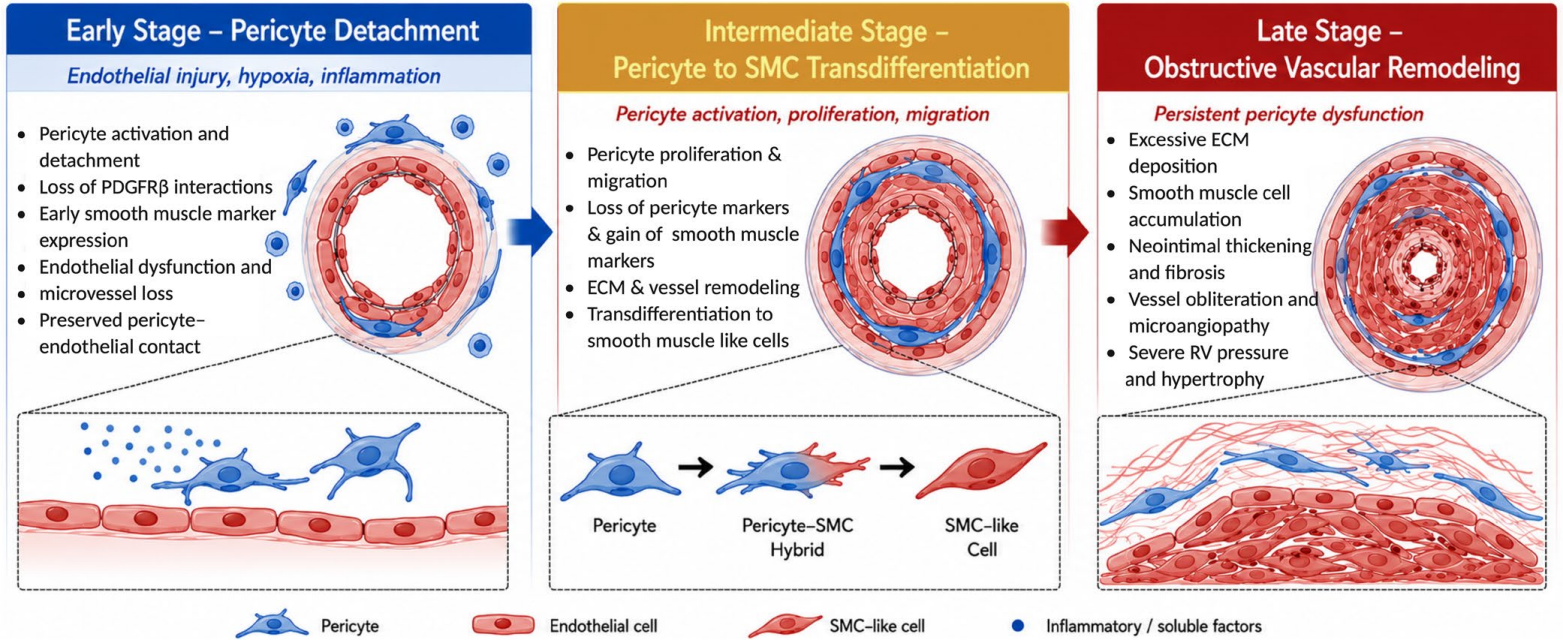
Proposed model of pericyte phenotypic transitions during pulmonary vascular remodeling. Pericytes contribute to pulmonary vascular remodeling through a progressive sequence of phenotypic and functional changes that evolve across disease stages. In the early stage, endothelial injury, hypoxia, and inflammation promote pericyte activation and detachment from the endothelial layer, resulting in disruption of PDGFRβ‐mediated endothelial–pericyte signaling, endothelial dysfunction, and microvascular loss. During this phase, pericytes remain in close proximity to endothelial cells but begin to lose their stabilizing vascular functions. In the intermediate stage, activated pericytes proliferate and migrate into the vascular wall, accompanied by downregulation of canonical pericyte markers and acquisition of smooth muscle cell (SMC)‐associated characteristics. This transitional state is associated with extracellular matrix (ECM) remodeling and the emergence of pericyte–SMC hybrid cells that contribute to medial thickening. In the late stage, persistent pericyte dysfunction drives excessive ECM deposition, accumulation of SMC‐like cells, neointimal formation, fibrosis, and progressive vessel obliteration. These structural changes culminate in microangiopathy, increased pulmonary vascular resistance, and right ventricular hypertrophy. Together, these stages illustrate a conceptual framework in which pericyte detachment, activation, and transdifferentiation contribute to the development of occlusive pulmonary vascular lesions and disease progression.

Pericytes contribute fundamentally to pathological vascular remodeling in PAH. Lineage tracing studies using NG2CreER transgenic mice demonstrate that pericytes expand significantly during chronic hypoxia‐induced pulmonary hypertension, migrating from capillaries to arterioles where they contribute to vessel muscularization. Histological analyses of PAH patient lung tissue reveal substantially increased pericyte numbers in distal pulmonary arteries, with excessive pericyte coverage correlating with medial thickening (Ricard et al. 2014). This abnormal accumulation represents an early feature of PAH pathogenesis, occurring before overt smooth muscle cell proliferation (Bordenave et al. 2020).

Pericytes from PAH patients exhibit intrinsic functional abnormalities, including enhanced migration, hyperproliferation, and increased capacity for differentiation into contractile smooth muscle‐like cells (Bordenave et al. 2020; Dabral et al. 2024). PAH pericytes display augmented proliferative and migratory responses even in the absence of pathological stimuli (Ricard et al. 2014; Dabral et al. 2024). Furthermore, they demonstrate impaired interactions with endothelial cells, failing to properly associate with endothelial tubes (Yuan et al. 2019, 2015; Klouda et al. 2023). This disrupted endothelial–pericyte crosstalk contributes to progressive microvascular loss and impaired vascular regeneration (Yuan et al. 2019, 2015; de Jesus Perez et al. 2012).

### 7.2 Pericyte‐to‐Smooth Muscle Cell Differentiation

A central mechanism by which pericytes drive PAH pathogenesis is through differentiation into smooth muscle‐like cells. Lineage tracing experiments demonstrate that NG2‐positive pericytes relocate from capillaries to arterioles during hypoxia‐induced pulmonary hypertension, where they co‐express smooth muscle actin and acquire contractile properties (Kim et al. 2024). Single‐cell transcriptional analyses identify Acta2‐positive pericytes that expand more than three‐fold during PAH development, exhibiting high expression of contractile markers (Cober et al. 2022). This pericyte‐to‐smooth muscle cell transition represents a major source of medial smooth muscle‐like cells in remodeled pulmonary arteries (Ricard et al. 2014).

The trans differentiation process is temporally regulated and biphasic: an early phase characterized by CXCL12‐dependent pericyte recruitment and proliferation, followed by a later phase in which canonical TGF‐β signaling drives pericyte differentiation into contractile cells (Bordenave et al. 2020; Garrison et al. 2023). Differentiated pericytes acquire functional contractile properties, exhibiting enhanced contractility and contributing to increased vascular tone (Kim et al. 2024, 2021).

### 7.3 Molecular Mechanisms and Signaling Pathways

Multiple interconnected signaling pathways orchestrate pathological pericyte behaviors in PAH. TGF‐β signaling plays a central role, with PAH pericytes overexpressing TGF‐β receptor II, rendering them hyperresponsive to TGF‐β ligands (Bordenave et al. 2020). Canonical TGF‐β signaling induces pericyte differentiation into smooth muscle‐like cells, enhances α‐SMA expression, and promotes pericyte proliferation and migration (Bordenave et al. 2020; Dabral et al. 2024).

PDGF signaling through PDGFR‐β drives pericyte hyperproliferation and migration. PDGF‐BB activates PI3K/AKT and ERK pathways, leading to forkhead box O3 (FoxO3) phosphorylation and nuclear exclusion, thereby promoting cell cycle progression (Dabral et al. 2024). HIF2α has emerged as a master regulator of pericyte transformation. HIF2α expression is markedly increased in PAH patient lung tissues, and pericyte‐selective HIF2α overexpression exacerbates pulmonary hypertension and right ventricular hypertrophy (Kim et al. 2024, 2021). HIF2α overexpressed pericytes exhibit greater contractility, impaired endothelial‐pericyte interactions, and enhanced transformation into smooth muscle‐like cells (Kim et al. 2024, 2021).

Wnt/PCP pathway regulates pericyte motility, polarization, and recruitment. PAH pericytes exhibit reduced expression of frizzled 7 and cdc42, key Wnt/PCP components, resulting in impaired pericyte‐endothelial interactions (Yuan et al. 2015). Loss of endothelium‐derived Wnt5a is associated with reduced pericyte recruitment and progressive small vessel loss (Yuan et al. 2019). Bone morphogenetic protein (BMP) signaling is critically dysregulated, with Acta2‐positive pericytes exhibiting Bmpr2 downregulation and Rbpms2 upregulation, inhibiting BMP signaling (Cober et al. 2022). Reciprocal changes in angiopoietin‐1 and angiopoietin‐2 expression favor endothelial cell activation and inflammation (Klouda et al. 2023; Cober et al. 2022).

Chemokine signaling through the CXCL12/CXCR7 axis drives early pericyte recruitment and expansion. PAH pericytes overexpress CXCR7, enhancing responsiveness to CXCL12 gradients and promoting migration to remodeling sites (Bordenave et al. 2020). Endothelial‐derived FGF‐2 and IL‐6 mediate increased pericyte coverage, with PAH endothelial cell conditioned media stimulating pericyte proliferation and migration (Ricard et al. 2014). Notch3 signaling is upregulated in pericytes during PAH. Loss of prolyl hydroxylase domain protein 2 in endothelial cells increases Notch3 and TGF‐β expression, leading to excessive pericyte coverage and differentiation into myofibroblasts and vascular smooth muscle cells (Wang et al. 2016).

### 7.4 Pericyte Migration, Proliferation, and Recruitment

In early hypoxia‐induced pulmonary hypertension, CXCL12‐dependent mechanisms drive pericyte expansion and recruitment to distal arterioles (Bordenave et al. 2020; Yuan et al. 2020). This is followed by local proliferation, with HIF2α‐positive cells accumulating in muscularized arterioles (Kim et al. 2024). Pericyte recruitment to endothelial cells depends critically on Wnt/PCP signaling. Endothelial‐derived Wnt5a triggers Wnt/PCP signaling through Vangl1 and ROR2 receptors on pericytes, promoting polarization and directional migration (Yuan et al. 2019, 2015). In PAH, loss of endothelial Wnt5a expression impairs pericyte recruitment, contributing to microvascular rarefaction (Yuan et al. 2019). CD13 activation mediates pericyte migration and proliferation, with CD13‐positive pericytes exhibiting a proliferative phenotype switch and decreased endothelial–pericyte interaction strength (James et al. 2023).

### 7.5 Pericyte Heterogeneity

Single‐cell transcriptomic studies reveal substantial pericyte heterogeneity. At least two specialized subtypes exist: Type 1 pericytes, which are quiescent, and Type 2 pericytes, which are lineage‐active and located near arterioles and capillaries (Klouda et al. 2025). In hypoxia‐induced pulmonary hypertension, Type 2 pericytes preferentially accumulate in arterioles, co‐express smooth muscle cell markers, and show increased vimentin expression, suggesting they are the primary subtype undergoing transition to smooth muscle‐like cells (Klouda et al. 2025).

### 7.6 Role in Different Stages of PAH Progression

Pericytes contribute to PAH in a stage‐dependent manner. In early stages, pericyte recruitment and proliferation predominate, driven by chemokine signaling and growth factors (Bordenave et al. 2020; Ricard et al. 2014; Kim et al. 2025). As PAH progresses, pericytes increasingly differentiate into smooth muscle‐like cells, driven by TGF‐β and HIF2α signaling (Garrison et al. 2023; Kim et al. 2024; Yuan et al. 2020; Kim et al. 2025). This mid‐stage transition involves pericyte migration from capillaries to arterioles, acquisition of contractile properties, and contribution to medial thickening. In advanced PAH, pericytes contribute to perivascular fibrosis through differentiation into myofibroblasts (Garrison et al. 2023). Late‐stage PAH is characterized by progressive microvascular loss due to impaired pericyte‐endothelial interactions and defective Wnt/PCP signaling (Yuan et al. 2015).

### 7.7 Therapeutic Implications and Targeting Strategies

The central role of pericytes in PAH has identified multiple therapeutic targets (Table 1). HIF2α inhibition represents a rational approach, with HIF2α inhibitors attenuating hypoxia‐induced pulmonary hypertension and reducing pericyte migration and vascular remodeling (Kim et al. 2024). CD13 blockade has emerged as a novel strategy, with a peptide blocking CD13 signaling (B‐CD13) significantly attenuating right ventricular systolic pressure and vascular remodeling while reverting high proliferation rates in pulmonary arterial smooth muscle cells from PAH patients (James et al. 2023).

C‐type natriuretic peptide (CNP)/cyclic guanosine monophosphate (cGMP)/FoxO3 pathway augmentation prevents pericyte proliferation, migration, and transdifferentiation by inhibiting PI3K/AKT and stabilizing FoxO3 (Dabral et al. 2024). Exogenous synthetic CNP and longer‐acting CNP analogs have shown efficacy in preclinical studies (Dabral et al. 2024). Wnt/PCP pathway restoration through augmentation of Fzd7 and cdc42 expression or Wnt5a supplementation may prevent microvascular loss and improve pericyte‐endothelial interactions (Yuan et al. 2019, 2015). TGF‐β and CXCR7/CXCR4 pathway inhibition represents a multi‐pronged approach to mitigate pericyte dysfunction (Garrison et al. 2023). PHD2/HIF‐2α/Notch3 axis modulation in endothelial cells represents a novel target for preventing pericyte‐mediated vascular remodeling (Wang et al. 2016).

### 7.8 Role of Pericytes in Right Ventricular Adaptation to PAH

Cardiac pericytes are increasingly recognized as important regulators of myocardial vascular homeostasis, tissue repair, and remodeling in cardiovascular disease. In the normal heart, pericytes maintain microvascular permeability, vascular tone, and endothelial cell survival (Dalkara et al. 2025; O'Farrell and Attwell 2014). Following myocardial infarction, pericytes contribute to the stabilization of newly formed vessels, regulate inflammatory cell recruitment, and can undergo phenotypic transitions toward myofibroblast‐like states that participate in scar formation and fibrosis (O'Farrell and Attwell 2014). Experimental studies have demonstrated that pericyte loss or dysfunction exacerbates microvascular rarefaction, impairs tissue perfusion, and worsens cardiac remodeling, whereas preservation of pericyte‐endothelial interactions supports angiogenesis and functional recovery. In chronic heart failure, pericyte abnormalities have been linked to capillary rarefaction, impaired coronary microcirculation, increased extracellular matrix deposition, and progressive ventricular dysfunction, culminating in a state of proangiogenic exhaustion that further undermines microvascular integrity (Katare et al. 2011; Simmonds et al. 2024). Collectively, these observations suggest that pericytes are critical determinants of the balance between adaptive vascular repair and maladaptive fibrotic remodeling in the diseased myocardium.

Although considerably less is known about pericytes in the right ventricle, emerging evidence suggests that these cells may play a central role in determining RV adaptation in PAH. Unlike the ischemic stress that drives left ventricular remodeling in MI and heart failure, the RV in PAH faces chronic pressure overload (Pullamsetti et al. 2026). Adaptive RV remodeling requires coordinated angiogenesis, preservation of capillary density, and maintenance of efficient endothelial–pericyte communication to meet the increased metabolic demands of pressure overload. Multiple preclinical PAH models have demonstrated decreased RV vascular density during RV failure, with capillary rarefaction linked to dysregulation of VEGF and angiopoietin signaling alongside fibrosis, inflammation, and metabolic reprogramming (Graham et al. 2018; Potus et al. 2015; Khassafi et al. 2023). By analogy with the failing left ventricle, RV pericytes may respond to chronic pressure overload and hypoxia by detaching from capillaries, acquiring fibrogenic or smooth muscle‐like identities, and amplifying the interstitial fibrosis and microvascular rarefaction that characterize maladaptive RV remodeling. Furthermore, impaired mechanotransduction in pressure‐overloaded RV pericytes could critically undermine the angiogenic response needed to sustain capillary density commensurate with RV hypertrophy, tipping the balance from compensation to decompensation. Elucidating the specific contributions of RV pericytes to these processes represents a compelling and largely uncharted frontier in PAH biology, with the potential to yield novel therapeutic strategies targeting both pulmonary vascular and right heart dysfunction.

### 7.9 Current Gaps and Future Directions

Despite significant advances, critical knowledge gaps remain. The precise molecular mechanisms governing pericyte heterogeneity and factors determining which pericyte subtypes undergo pathological transformation require further elucidation. The temporal dynamics of pericyte dysfunction in human PAH remain poorly characterized, with most mechanistic insights derived from animal models. The reversibility of pericyte differentiation and the potential for pericyte‐targeted therapies to reverse established vascular remodeling remain uncertain. Future research should include comprehensive single‐cell and spatial transcriptomic analyses of human PAH lung tissue to map pericyte heterogeneity; development of pericyte‐specific genetic models in large animal models; investigation of sex‐specific differences in pericyte biology; exploration of metabolic reprogramming in PAH pericytes; clinical trials of pericyte‐targeted therapies; and development of imaging biomarkers to assess pericyte coverage non‐invasively. Addressing these gaps will be essential for translating mechanistic understanding into effective therapeutic strategies.

## 8 Lung Cancer and Tumor Angiogenesis

Lung pericytes play critical roles in tumor angiogenesis and the tumor microenvironment, serving as key regulators of vascular function and tumor progression in lung cancer. These perivascular cells contribute to tumor growth through multiple mechanisms, including regulation of angiogenic signaling pathways, modulation of vascular stability, and direct interactions with tumor cells. Recent research has identified several molecular pathways through which pericytes influence lung cancer pathogenesis, with particular emphasis on their role in promoting tumor angiogenesis and creating a permissive microenvironment for tumor expansion.

### 8.1 CD248‐Wnt Signaling Axis in Tumor Angiogenesis

CD248, a pericyte‐specific marker, has emerged as a crucial regulator of tumor angiogenesis in lung cancer through its modulation of Wnt signaling. CD248 de‐represses Wnt/β‐catenin signaling in pericytes by interacting with Wnt pathway repressors IGFBP4 and LGALS3BP, leading to increased expression of angiogenic factors OPN and SERPINE1 (Hong et al. 2022). This CD248‐Wnt signaling axis enhances angiogenesis and promotes lung cancer growth, with loss of CD248 or Wnt inhibition resulting in reduced tumor volume and impaired vessel functionality (Hong et al. 2022).

Administration of β‐catenin inhibitors mimics CD248 loss effects, blocking tumor growth and suggesting a viable therapeutic strategy (Hong et al. 2022). In Cd248‐deficient pericytes, decreased OPN and SERPINE1 expression reduces pericyte proliferation and their ability to promote endothelial cell tube formation (Hong et al. 2022). Loss of CD248 also results in lower tumor vessel density and functionality, suppressing angiogenesis and lung cancer growth in orthotopic models (Hong et al. 2022). These findings establish the CD248‐Wnt signaling‐angiogenic factors axis in pericytes as a potential therapeutic target for lung cancer treatment.

### 8.2 Pericyte Recruitment and Vascular Normalization

Pericyte recruitment and function in the tumor microenvironment are dynamically regulated by tumor‐derived factors. In lung adenocarcinoma, tumor ischemia and hypoxia following anti‐angiogenesis therapy upregulate CCL28 expression in cancer cells, which recruits pericytes and metabolically reprograms them [67]. This CCL28‐mediated recruitment promotes vascular normalization through the retinoic acid (RA)/RXRα/ANGPT1 pathway, with CCL28 modifying retinoic acid metabolism and increasing ANGPT1 expression via RXRα in pericytes, thereby enhancing endothelial cell stability (Chen et al. 2024).

This mechanism suggests that pericyte recruitment can paradoxically promote vascular normalization after anti‐angiogenesis therapy, potentially enhancing the efficacy of combination treatments including chemotherapy, radiotherapy, targeted therapy, and immunotherapy (Chen et al. 2024). Understanding the CCL28/RA/RXRα/ANGPT1 pathway could provide molecular markers for guiding clinical combination therapy between anti‐angiogenesis treatment and other therapeutic modalities (Chen et al. 2024).

### 8.3 Tumor‐Derived Perivascular‐Like Cells

Tumor‐derived perivascular‐like cells exhibit distinct pathological characteristics that contribute to tumor progression and immune evasion. In non‐small cell lung cancer (NSCLC), tumor‐derived perivascular‐like cells demonstrate increased PD‐L1 expression, elevated IL‐6 secretion, and higher α‐SMA levels compared to normal lung pericytes (Bichsel et al. 2017). These cells promote vascular leakage in perfusable microvasculature models, indicating functional abnormalities that may facilitate tumor cell dissemination and metastasis (Bichsel et al. 2017).

The increased PD‐L1 expression on tumor‐associated pericytes suggests a role in immune evasion, potentially contributing to the immunosuppressive tumor microenvironment. This finding has important implications for immunotherapy, as pericytes may represent an additional cellular target for immune checkpoint blockade beyond tumor cells themselves. The elevated IL‐6 secretion by tumor‐associated pericytes also suggests these cells contribute to the inflammatory milieu that supports tumor growth and progression.

### 8.4 Notch3‐Mediated Stromal Interactions

Single‐cell analysis has identified NOTCH3‐mediated interactions between stromal cells, including pericytes, that promote microenvironment remodeling and invasion in lung adenocarcinoma (Xiang et al. 2024). These findings highlight the importance of pericyte interactions with other stromal cell types in shaping the tumor microenvironment and facilitating tumor progression. The NOTCH3 pathway in pericytes may coordinate multiple aspects of stromal remodeling, including ECM deposition, angiogenesis, and immune cell recruitment (Li et al. 2009).

### 8.5 Therapeutic Implications

Therapeutic targeting of pericytes in lung cancer represents a promising strategy for improving treatment outcomes (Table 1). The CD248‐Wnt signaling axis provides multiple intervention points, with both CD248 targeting and β‐catenin inhibition showing efficacy in preclinical models (Hong et al. 2022). Strategies to modulate this pathway could reduce tumor angiogenesis and slow tumor growth.

Understanding the CCL28/RA/RXRα/ANGPT1 pathway provides opportunities for enhancing the efficacy of anti‐angiogenesis therapy through promotion of vascular normalization (Chen et al. 2024). This approach could improve drug delivery and enhance the effectiveness of chemotherapy, radiotherapy, and immunotherapy by normalizing the chaotic tumor vasculature.

Additionally, the identification of PD‐L1 expression and IL‐6 secretion by tumor‐associated perivascular‐like cells suggests these cells as potential targets for immunotherapy and anti‐inflammatory interventions (Bichsel et al. 2017). Combination strategies targeting both tumor cells and tumor‐associated pericytes may be necessary to achieve optimal therapeutic outcomes. These pericyte‐focused therapeutic strategies aim to normalize tumor vasculature, reduce angiogenesis, enhance immune surveillance, and improve the efficacy of existing cancer treatments.

## 9 Pneumonia and Lung Infections

Lung pericytes play essential roles in the pathogenesis and resolution of pneumonia and lung infections, serving as critical mediators of vascular barrier function, immune responses, and tissue repair. These perivascular cells respond dynamically to pathogen challenge, exhibiting remarkable plasticity in their functional states during infection. Recent research has revealed that pericytes not only maintain structural integrity of the pulmonary microvasculature during infection but also actively participate in immune signaling and inflammatory responses, positioning them as key regulators of infection outcomes.

### 9.1 Pericyte Responses in Viral Lung Injury

During viral lung injury, pericytes demonstrate significant angiogenic activity that influences vascular permeability and endothelial integrity. Studies of experimental viral lung injury have shown that lung pericytes promote endothelial network formation and modulate monolayer permeability through angiopoietin‐like 4 (ANGPTL4) (Hung and Altemeier 2022). ANGPTL4 secreted by pericytes affects endothelial cell behavior, with knockdown of ANGPTL4 hypothesized to be protective in acute viral lung injury by promoting endothelial integrity and decreasing microvascular leak during both injury and resolution phases (Hung and Altemeier 2022).

This mechanism highlights the dual nature of pericyte responses during infection, where their activation can contribute to both pathological vascular leak and subsequent repair processes. Pericyte‐like cells exposed to injured bronchoalveolar lavage fluid show activation of immuno‐inflammatory, cell migration, and angiogenesis‐related pathways, while processes associated with tissue development and remodeling are down‐regulated. These cells exhibit enhanced invasiveness, suggesting increased migration capacity that may facilitate their participation in tissue repair (Hung et al. 2021).

### 9.2 Pericyte Dysfunction in Sepsis and Bacterial Pneumonia

In sepsis and bacterial pneumonia, pericyte dysfunction represents a critical pathological mechanism contributing to vascular instability and organ damage. The transcription factor Fli‐1 governs pericyte dysfunction in murine models of sepsis, with Fli‐1 upregulation in lung pericytes leading to pericyte loss, increased vascular leak, and reduced survival. Mechanistically, Fli‐1 promotes pericyte pyroptosis, a form of inflammatory cell death, during sepsis‐induced lung injury (Li et al. 2018).

Knockout of Fli‐1 prevents pericyte loss and mitigates sepsis‐induced pericyte pyroptosis, while disruption of Fli‐1 expression by small interfering RNA inhibits LPS‐induced inflammatory cytokine and chemokine production in cultured lung pericytes (Li et al. 2018). These findings establish Fli‐1 as a key regulator of pericyte responses to bacterial infection and sepsis and suggest that targeting Fli‐1 could preserve pericyte function and improve outcomes in severe bacterial infections.

### 9.3 Pericytes as Mediators of Inflammation

Pericytes function as active mediators of inflammation during lung infections, responding to pathogen‐associated molecular patterns and orchestrating immune cell recruitment. Lung pericytes possess the capacity to respond to PAMPs and DAMPs, mediating inflammation through leukocyte trafficking and cytokine signaling (Rayner et al. 2023). Their involvement in the initial inflammatory process during infection has been increasingly appreciated, with pericytes exhibiting an inflammatory phenotype during organ injury (Rayner et al. 2023).

Human lung pericytes express functional toll‐like receptors (TLR2, TLR4, TLR6), and stimulation of these receptors leads to chemokine expression (Wilson et al. 2018). Necrotic cell lysate triggers NLRP3 inflammasome assembly and robust chemokine induction in pericytes (Wilson et al. 2018). This inflammatory activation of pericytes contributes to the host response to infection but may also drive excessive vascular inflammation, as observed during SARS‐CoV‐2 infection where pericytes may contribute to COVID‐19‐associated vascular pathology (Rayner et al. 2023).

The multifaceted roles of pericytes extend beyond acute inflammation to encompass tissue repair, with these cells contributing to structural repair and immune dynamics during recovery from infection (Schnapp 2025). Understanding the balance between protective and pathological pericyte responses is critical for developing therapeutic strategies that enhance pathogen clearance while minimizing tissue damage.

### 9.4 Therapeutic Targeting in Lung Infections

Therapeutic targeting of pericytes in pneumonia and lung infections offers potential strategies for improving outcomes (Table 1). ANGPTL4 represents a promising therapeutic target, with its inhibition potentially promoting endothelial integrity and reducing microvascular leak during viral lung injury (Hung and Altemeier 2022). Modulating ANGPTL4 activity could help maintain vascular barrier function while allowing appropriate immune responses to proceed.

Targeting Fli‐1 in sepsis presents another therapeutic avenue, as Fli‐1 knockout improves survival and prevents pericyte loss and vascular leak in experimental models (Li et al. 2018). Small molecule inhibitors or RNA‐based therapeutics targeting Fli‐1 could preserve pericyte function and reduce vascular instability in severe bacterial infections and sepsis.

A comprehensive understanding of pericyte plasticity and heterogeneity during infection could facilitate the development of novel treatments that preserve pericyte function while limiting pathological activation (Schnapp 2025). Future therapeutic strategies may focus on maintaining pericyte‐endothelial communication, preventing pericyte loss through inhibition of pyroptosis, and modulating pericyte inflammatory responses to optimize both pathogen clearance and tissue preservation during lung infections. The development of such targeted therapies could significantly improve outcomes in pneumonia, sepsis, and viral lung infections, including pandemic respiratory viruses.

## 10 Future Directions and Therapeutic Opportunities

Pericytes have emerged as attractive therapeutic targets given their central roles in angiogenesis, vascular stabilization, and tissue repair. Experimental studies in animal models have explored the use of pericyte‐based cell therapies to promote vascular recovery in conditions such as retinal vasculopathy (Mendel et al. 2013) and myocardial infarction (Alvino et al. 2018; Avolio et al. 2015; Chen et al. 2013), with encouraging results. Recent encouraging results with a matrix graft cellularized with cardiac pericytes in a swine model of pulmonary artery reconstruction highlight that biomaterials capable of supporting pericyte survival and function may represent a novel therapeutic avenue (Alvino et al. 2021). After reviewing the roles of pericytes across a spectrum of lung disorders, it is now possible to begin identifying opportunities where modulation of pericyte biology may prove beneficial. The following sections highlight emerging concepts and potential strategies that could guide the development of pericyte‐based therapies for chronic lung diseases.

### 10.1 Integration of Signaling Pathways

A central finding is the extensive integration and crosstalk among signaling pathways in pericytes. Rather than operating as independent modules, pathways such as PDGF‐BB/PDGFRβ, TGFβ, Notch, Wnt, and mechanotransduction form interconnected networks that coordinately regulate pericyte phenotype and function. The Notch1/PDGFRβ/ROCK1 axis exemplifies this integration, where Notch signaling modulates growth factor receptor activity and downstream cytoskeletal regulation (Wang et al. 2019). Similarly, the HIF‐1α/Notch1/PDGFRβ/YAP1/Twist1 axis demonstrates convergence of hypoxia signaling, growth factor pathways, and mechanotransduction (Wang et al. 2021). This pathway integration has important therapeutic implications. Single‐pathway inhibition may be insufficient to achieve desired outcomes, as compensatory signaling through alternative pathways can maintain pathological pericyte phenotypes. Multi‐targeted approaches, such as nintedanib, which inhibits PDGFRβ, FGFR, and VEGFR (Leonard‐Duke et al. 2025), may be more effective. Future therapeutic development should consider combination strategies that target multiple nodes in integrated signaling networks.

### 10.2 Pericyte Heterogeneity and Functional Specialization

Emerging evidence from single‐cell and spatial transcriptomic studies reveals substantial heterogeneity among lung pericyte populations (Klouda et al. 2025; Travaglini et al. 2020). Specialized pericyte subtypes exist in different regions of the pulmonary vasculature, with distinct molecular signatures and functional properties (Klouda et al. 2025). The identification of β‐III tubulin as a marker of an anti‐fibrotic pericyte state (Sato et al. 2025) suggests that pericyte subpopulations may have opposing roles in disease pathogenesis. Understanding this heterogeneity is critical for developing targeted therapies. Interventions that globally deplete or activate all pericytes may have unintended consequences if distinct subpopulations serve protective versus pathological functions. Future research should focus on identifying markers that distinguish beneficial from detrimental pericyte subsets and developing strategies to selectively modulate specific populations. Spatial transcriptomics and lineage tracing studies will be essential tools for mapping pericyte heterogeneity and tracking phenotypic transitions in disease. An important caveat, however, is that it remains largely unclear whether phenotypic transitions such as pericyte‐to‐myofibroblast or pericyte‐to‐SMC‐like differentiation are reversible once disease is established. The existence of distinct pericyte subtypes (e.g., Type 1 and Type 2) implies that the mechanisms governing homeostatic versus pathogenic pericyte states are not yet fully understood, and the markers needed to reliably distinguish protective from deleterious subpopulations in vivo remain incompletely defined. Defining molecular markers that reliably distinguish beneficial from deleterious pericyte states and understanding the mechanisms governing fate decisions will be critical prerequisites for the development of selective pericyte‐directed therapies.

### 10.3 Mechanotransduction as a Therapeutic Target

The recognition that mechanical forces profoundly influence pericyte phenotype opens new therapeutic avenues. In pulmonary fibrosis, the feed‐forward loop where pericyte‐derived ECM increases tissue stiffness, which in turn drives further pericyte activation through mechanotransduction (Sava, Ramanathan, Dobronyi, Peng, et al. 2017), suggests that targeting mechanosensitive pathways could break this pathological cycle. YAP/TAZ, MKL‐1/MRTFA, and integrin signaling represent potential intervention points. Nintedanib's ability to reverse the mechanical properties of fibrotic matrices through promotion of MMP activity in pericytes (Sava, Ramanathan, Dobronyi, et al. 2017) demonstrates proof‐of‐concept for this approach. Future therapeutic development could focus on more selective modulators of mechanotransduction pathways or on strategies to modify the mechanical properties of the lung ECM. Understanding how substrate stiffness, shear stress, and cyclic stretch are sensed and transduced into biochemical signals in pericytes will be essential for this effort.

### 10.4 Epigenetic Regulation and Cellular Reprogramming

The discovery that epigenetic mechanisms, including long non‐coding RNAs and histone modifications, regulate pericyte phenotypic transitions (Wang et al. 2023) suggests opportunities for therapeutic reprogramming of pericytes. GAS5‐mediated recruitment of KDM5B to demethylate the PDGFRα/β promoter (Wang et al. 2023) demonstrates that pericyte phenotype can be modulated through epigenetic mechanisms. MicroRNA‐based therapies, including exosomal miR‐107 (Wang et al. 2021) and miR‐146b‐5p (Shuai et al. 2022), represent another approach to cellular reprogramming. Future research should comprehensively map the epigenetic landscape of pericytes in different disease states and identify key regulatory elements that control phenotypic transitions. CRISPR‐based epigenome editing could enable precise modulation of pericyte phenotype without permanent genetic modification. Delivery of therapeutic RNAs or epigenetic modifiers specifically to pericytes will require development of targeting strategies, potentially leveraging pericyte‐specific markers such as PDGFRβ or CD248. While these findings support the concept of therapeutic pericyte epigenetic reprogramming, it remains uncertain whether established pathogenic pericyte phenotypes can be fully reversed in vivo. Most studies to date have focused on prevention or attenuation of disease progression rather than restoration of normal pericyte identity after advanced tissue remodeling has occurred. Future studies employing lineage tracing, single‐cell multi‐omics, and longitudinal disease models will be necessary to determine the reversibility of pericyte state transitions and to identify windows of therapeutic opportunity.

### 10.5 Pericytes in Emerging Lung Diseases

The COVID‐19 pandemic has highlighted the importance of pericytes in viral lung injury and vascular inflammation (Rayner et al. 2023). Pericytes may drive vascular inflammation during SARS‐CoV‐2 infection, contributing to the microvascular thrombosis and endothelial dysfunction observed in severe COVID‐19. Climate change and environmental exposures are driving increases in lung diseases including asthma, COPD, and lung cancer. The role of pericytes in these conditions, particularly in response to environmental stressors such as air pollution, oxidative stress, and allergens, deserves further investigation. The finding that ROS‐induced endothelial stress contributes to pulmonary fibrosis through pericytes and Wnt signaling (Andersson‐Sjöland et al. 2016) suggests that environmental oxidative stress may influence pericyte function.

## 11 Conclusions

Lung pericytes have emerged as critical regulators of pulmonary vascular homeostasis and key contributors to diverse lung diseases. This comprehensive review has synthesized current knowledge on the molecular mechanisms governing pericyte function and the multifaceted roles of pericytes in major pulmonary diseases have been examined in detail. The translation of mechanistic insights into clinical therapies faces several challenges, including the need for pericyte‐specific delivery systems, identification of optimal timing for interventions, and development of biomarkers for patient stratification. Addressing these priorities will require multidisciplinary collaboration among cell biologists, molecular scientists, bioengineers, immunologists, and clinicians, as well as the continued development of advanced experimental platforms (e.g., organoids, lung‐on‐a‐chip systems, and humanized animal models) to translate mechanistic insights into clinically actionable strategies.

## Author Contributions

A.B. and S.A. conducted the literature review, synthesized the evidence, and drafted the initial manuscript. V.A.J.P. conceived the overall framework and scope of the review, contributed to interpretation and integration of the literature, and critically revised the manuscript for important intellectual content. All authors contributed to manuscript editing, approved the final version, and agree to be accountable for the content of the work.

## Funding

This work was supported by an R01HL139664, R01HL134776, R01HL59886, R01HL160018, 5R01HL172449‐02 to V.A.J.P.

## Conflicts of Interest

The authors declare no conflicts of interest.

## Data Availability

Data sharing not applicable to this article as no datasets were generated or analysed during the current study.
